# An adaptive spinal-like controller: tunable biomimetic behavior for a robotic limb

**DOI:** 10.1186/1475-925X-13-151

**Published:** 2014-11-20

**Authors:** Filip Stefanovic, Henrietta L Galiana

**Affiliations:** Department of Biomedical Engineering, McGill University, 3775, rue University, Room 316, Montréal, QC H3A 2B4 Canada

**Keywords:** Adaptive control, Arm motions, Goal-oriented reaching, Tunable regulator, Biomimetic

## Abstract

**Background:**

Spinal-like regulators have recently been shown to support complex behavioral patterns during volitional goal-oriented reaching paradigms. We use an interpretation of the adaptive spinal-like controller as inspiration for the development of a controller for a robotic limb. It will be demonstrated that a simulated robot arm with linear actuators can achieve biological-like limb movements. In addition, it will be shown that programmability in the regulator enables independent spatial and temporal changes to be defined for movement tasks, downstream of central commands using sensory stimuli. The adaptive spinal-like controller is the first to demonstrate such behavior for complex motor behaviors in multi-joint limb movements.

**Methods:**

The controller is evaluated using a simulated robotic apparatus and three goal-oriented reaching paradigms: 1) shaping of trajectory profiles during reaching; 2) sensitivity of trajectories to sudden perturbations; 3) reaching to a moving target. The experiments were designed to highlight complex motor tasks that are omitted in earlier studies, and important for the development of improved artificial limb control.

**Results:**

In all three cases the controller was able to reach the targets without a priori planning of end-point or segmental motor trajectories. Instead, trajectory spatio-temporal dynamics evolve from properties of the controller architecture using the spatial error (vector distance to goal). Results show that curvature amplitude in hand trajectory paths are reduced by as much as 98% using simple gain scaling techniques, while adaptive network behavior allows the regulator to successfully adapt to perturbations and track a moving target. An important observation for this study is that all motions resemble human-like movements with non-linear muscles and complex joint mechanics.

**Conclusions:**

The controller shows that it can adapt to various behavioral contexts which are not included in previous biomimetic studies. The research supplements an earlier study by examining the tunability of the spinal-like controller for complex reaching tasks. This work is a step toward building more robust controllers for powered artificial limbs.

## Background

Adaptive spinal-like controllers have recently shown to be versatile regulators for sensory-based limb control. In particular, investigators have shown that a substantial contribution can be made by the motor centers in the spinal cord during motor responses and execution [1–8]. As such, it is assumed that they can be used to develop more life-like controllers for artificial limbs. Here, we aim to investigate the adaptive spinal-like controller (ASC) [9] on a robotic platform and test its proposed applicability for the control of a robotic limb, with particular focus on its tunability, using various complex reaching tasks. It is also of particular interest for this research to demonstrate that a linear non-biological robot arm can replicate biological-like reaching behaviors of a non-linear musculoskeletal system using this modified ASC.

Earlier studies of volitional reaching mechanisms relied mainly on an implicit dominant contribution from higher CNS motor centers. Those controllers typically consign the spinal cord to a subordinate role in the execution of pre-planned motor behavior [10–16]. This is based on evidence of activity in the higher CNS *before* motion onset, which suggests that motor actions are pre-planned centrally and then executed as context-dependent movements. Such movement planning strategies can provide exceptional similarities to biological data for several tasks, particularly regarding the characteristic speed profiles and smoothness of movement trajectories. This approach to motor control advocates that the cerebellum [11, 12] and the motor cortex function for the most part independently from spinal motor centers. However, it is unclear how reflex pathways [17–21] or central pattern generators [1, 2] would interact with these fundamental structures. Others suggest that rather than pre-planning motion kinematics or dynamics, an inherent equilibrium in the mammalian muscular system guarantees smooth motion [14, 15, 22]. That is, as the length-tension properties of the muscles in a limb change, the shifting equilibrium position itself defines a movement trajectory to reach a goal. This theory advocates that the properties of spinal reflex circuits can be exploited by the brain to simplify movement problems. In any case, the spinal cord is still regarded as a medium for higher-level motor planning, but its natural characteristics can influence the execution of the motor task and reduce the complexity of necessary central interventions.

More recently, physiological studies have shown that spinal motor centers include complex programmability and computational capacity [1–5]. For example, Tresch et al.’s [2] work examined spinal motor networks in vertebrates. They demonstrated that these networks link muscles with shared pathways that elicit complex movements even when separated from higher CNS function. Additionally, spinal motor centers demonstrate an inherent intrasegmental coupling for complex motor tasks [23, 24]. This suggests that some sensory-based motions could originate in the spinal-cord itself, as opposed to relying solely on central commands. Ultimately, they argue that it is more likely for a central pattern generator (CPG) to exist in the spinal-cord, while strongly coupled brain and spinal motor areas would generate a volitional motor task. McCrea and Rybak went on to suggest that the CPG may be a two-level system which includes a rhythm generator and a pattern formation circuit [25]. The rhythm generator would maintain period and phase of a motor oscillation while the pattern formation circuit consists of spinal interneurons and motoneurons for muscle recruitment. These pattern formation circuits are reminiscent of spinal reflex topologies. These complex systems responsible for coordinated muscle activity patterns have long been believed to be responsible for sensory based neuromuscular response, but investigations into their role for volitional movement control have only recently begun.

Interestingly, it has been shown that these spinal circuits could possibly be modulated by downstream projections from reach related neurons in the superior colliculus [26–28]. It is known that a major efferent pathway from the superior colliculus is to the cervical spinal cord for coordinated motor control. It is assumed that the reach related neurons which discharge in the underlying layers project downstream to the spinal motor centers much like the discharges to the visual areas [29, 30].

Kurtzer and colleagues [31] also demonstrated that spinal reflex-based motor centers can exhibit intelligent motor functions that resemble internal models. In particular, they demonstrated that reflex responses to perturbations changed in order to account for limb geometry, applied torques, and joint motion. This is also related to the anticipatory discharges in Renshaw cells which are known to play a role in estimating sensory feedback – i.e., an expected efference copy [32, 33]. These reflex motor circuits in the spinal cord are thus assumed to have significant programmability for volitional motor tasks, as leveraged by our previous work [9].

Raphael, Tsianos and Loeb [5] recently demonstrated how a spinal-like regulator (SLR) based on known spinal reflexive circuitry can perform 2 DOF *wrist* movements. They demonstrated that complex motions were easily obtained without exhaustive pre-planning and that their spinal-like circuit ensured stable movements. Their investigations also demonstrated that the SLR performs exceptionally well in motor learning tasks. The same research group recently extended their work to include multi-segment symmetric planar reaching systems to demonstrate how spinal-like circuits can also facilitate stabilization for redundant musculature [34, 35]. These important findings were also supported by a similar system defined in [9]. Equally significant, their spinal-like models show that motor tasks can be learned over time and that the learning changes based on the mode of the controller. Clearly, these findings are significant for not only describing modern interpretations of neuromuscular control of movement, but also in defining vastly improved systems for rehabilitation and neuroprosthetic limbs [36].

We use the ASC defined in [9] to test applicability and biomimetic control for a multi-joint robot limb using bi-segmental planar reaching tasks over various ranges in the workspace. The novelty will show that programmability in the ASC enables distinct spatial and temporal changes to be independently defined to create kinematic scalability for limb movement tasks without solving/optimizing inverse kinematics models. The controller described in our earlier works for biological simulations [9] is revised to represent a two-link simulated robot *arm* (linear actuators). Earlier it was shown that the controller can perform biological-like reaching motions without planning using non-linear muscle models. Here, it will be demonstrated that the same controller can perform biological-like tasks using a non-physiological robotic platform, and a powerful tunability that scales movements. By demonstrating that the ASC can define biological-like reaching behaviors using a non-physiological system, it is believed that it can be used to develop more life-like artificial limb controllers. In this article, we use the ASC to explore two hypotheses: (1) *Spatial scaling*: trajectory curvature can be tuned using the inherent spinal gains rather than the normally assumed pre-planned trajectories or kinematics in cortical areas; (2) *Temporal scaling*: bell-shaped speed profiles of the end-point along its trajectory are an intrinsic property of the ASC that can be manipulated to define a desired amplitude or shape (e.g., scaling movement amplitude and speed).

Motions are simulated in Matlab using a model of a robotic arm whose unusual segment geometry is intentionally chosen to test the controller’s kinematic tunability – this makes scaling more difficult than in symmetrical limbs such as in our previous studies [9] or in [34]. This simulation study of a robot apparatus provides a simplified test bed that allows the behaviors of the controller to be examined without the additional complexities of a non-linear system. It should not be assumed that this robot arm, and its properties, will be used as a prosthetic limb. We demonstrate that the ASC is capable of performing complex motions that earlier biomimetic controllers fail to accomplish including tracking moving targets, adapting to dynamic environmental conditions, and an ability to maintain end-point accuracy at high speeds [10, 14]. As a result, it provides a novel alternative for the development of artificial arm controllers during volitional reaching tasks that require feedback, intrinsic stabilizing effects, feasibility in a variety of movement contexts, and programmability for motion goals. This work is a step toward developing a prosthetic limb regulated by the ASC.

## Methods

### Robot apparatus

In order to test the controller using simulations, we first parametrically model an Adept Cobra s350 SCARA robotic arm (Adept Technology Inc., USA) as a platform for feasibility tests. This robot is typically regulated by a SmartController CX controller that is programmed using the proprietary Adept A + language. Control of the robot is normally limited to an extrinsic coordinate space (e.g., selection of desired end-point coordinates) and does not allow for the user to specify the motor inputs; however we bypass the safety protocols in the A + software in order for the ASC to be able to directly apply voltage to the motors. In this mode, a voltage applied to the robot motors is converted into a motor encoder position change. For example, when ~0.5 volts are applied to the motor for 16 ms, the encoder position moves by 850 bits – and there are 9104 encoder bits per degree. All other specifications relating to motor capabilities, and the SmartController CX can be found in the documentation provided by the manufacturer.

**Figure 1:**
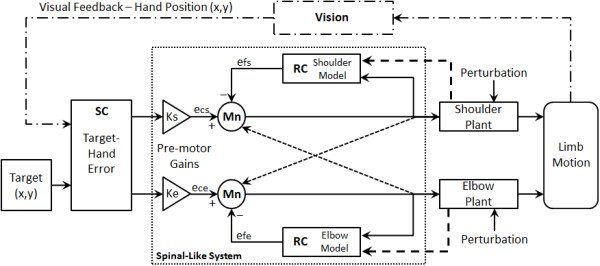
**The adaptive spinal-like controller for a two joint planar robot arm.** Here, *e*_*cs*_ and *e*_*ce*_ are primary shoulder and elbow motor drives based on weighted visual error from the superior colliculus (SC); *e*_*fs*_ and *e*_*fe*_ are ‘efference copy’ motor predictions based on expected plant behavior from the Renshaw Cells (RC), and K_s_ and K_e_ are gains which can internally modify the controller signals. The combined input to the motorneuron (Mn) drives the motors for each segment. Direct intrasegmental connections via interneurons (dashed) are not used in this study since the motors rotate bidirectionally and the dynamic brakes negate the interaction torques. Here, the internal models imbed the combined effect of motor dynamics, sensory-derived mechanical perturbations and internal controller signals - hence predicted states can reflect and compensate for mechanical perturbations. Refer also to Figure 2.

The robot arm is a two-link, 2-DOF system whose joints (shoulder and elbow) rotate uniformly in both directions. For our experiments, the robot’s servomotors minimize the effects of interaction torques [37–39] between the segments (Figure 1, dashed lines) since they prevent involuntary motion in the motors/segments. Note that this is unlike a real multi-muscle system under load where interaction torques are a significant component of the kinematics, and the motors themselves (muscles) are highly non-linear. We examined this as well as the effect of feedback delays in earlier work [9] with no significant changes in performance. Additionally, the distance from the shoulder to the elbow, *l*_s_, is 12.5 cm, and the distance from the elbow to the wrist, *l*_e_, is 22.5 cm.

We employ standard system identification (SID) methods discussed in earlier works [40, 41] in order to generate parametric model approximations relating input voltage levels to motor positions (i.e., limb angles). In short, this process involves applying a zero-mean white noise signal (i.e., voltage) as input to the plant, recording the resulting output from the plant (i.e., encoder position), and then defining a transfer function relating input/output. By applying voltages to both motors during identification, the multi-segment dynamics are also represented and are included in the models. The resulting transfer function provides a parametric approximation of the system – eqs. (6) and (7). Thus, the identified models provide an approximation of the input voltage vs. output that achieves a desired rotation as well as the dynamics of the interacting segments associated with these changes. Consequently, it should be understood that the kinematics/dynamics of the hardware system are included in these parametric models. The robot arm is modeled at 62.5 Hz. These parametric models will be used to approximate the “Models” and “Plants” of the control schematic during simulations (Figure 1) while visual feedback is provided by Matlab. To simplify, we assume the Plant (i.e., motor unit) and Model (i.e., efference copy or internal approximation) are identical at each joint/motor. Note that the robot motors can receive servomotor input voltages from 0 ± 10 volts. However, the simulated robot arm is restricted to have a normal operating range of 0 ± 2 V, so that the rotational speeds remain close to those of human movements (approx. 60-80 deg/s) [42]. Accordingly, the parameter set in all simulations ensures that all unperturbed motor drives remain in the 0 ± 1 V range (Table 1).

**Table 1:** **Default parameter set for simulations**

***Parameter***	***Value***
K_e_	1
K_s_	1
l_s_	12.5 cm
l_e_	22.5 cm
Matlab sampling rate	62.5 Hz
Non-perturbation motor drive	0 ± 1 Volts
Perturbation	1 Volt

Note that this is a high-power robot that can reach its maximum speed in a very short time leading to un-human like speed profiles (e.g., near-instantaneous speed changes). Thus, the dynamics are low pass filtered to limit the bandwidth to the human-like range. For example, this is implemented in the simulation by using a dynamic ratio, *i*^*2*^/30^2^ on the initial internal spatial error; where *i* is the sample number from samples 1 to 30. Then, after the first 30 samples of the simulation the dynamic ratio is held at 1. Note that this does not affect the behavior of the controller, only the initial dynamics due to sudden changes in voltage. In any case, this is not a limitation caused by the biomimetic controller or its implementation since the results match those in [9], rather the robot apparatus is too powerful and we wish to examine reaching in the dynamic range seen in human data.

### Controller topology and its simulation

**Figure 2:**
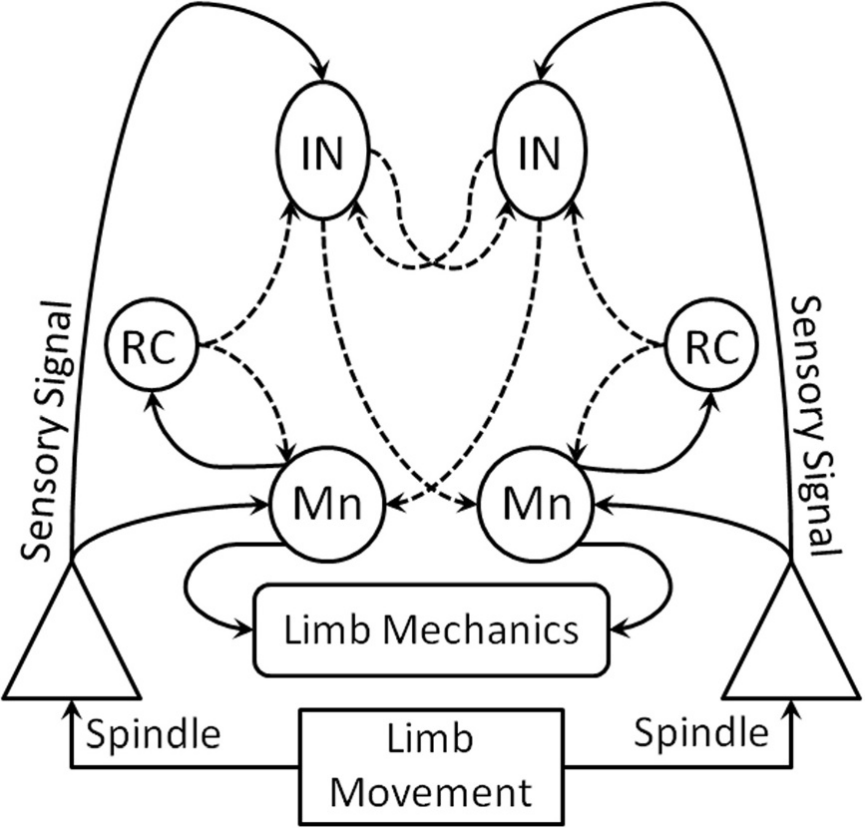
**Spinal-like motor circuit.** Proprioceptive stimuli from spindles produce sensory signals that innervate interneurons (IN) or motoneurons (Mn) directly. Interneurons provide inhibitory and excitatory pathways to both antagonist/agonist Mn and IN while Renshaw Cells (RC) provide an efference copy to the circuit. The spinal-like circuit demonstrates cross-connections and symmetry while activating antagonist Mn groups.

Figure 1 demonstrates the controller design based on the spinal topology from Figure 2. Since the motors are linear and bi-directional, the agonist-antagonist pair from [9] is reduced to a single lumped unit for each segment. In addition, spinal efference models of the shoulder and elbow dynamics are represented by the Renshaw cells (RC) as per their role in estimating limb dynamics [32, 33]. We assume that feedback-based efference models of the limb dynamics contribute to the involuntary (reflexive) motor intelligence presented in [31] – for the schematic we assume positive feedback, but negative feedback can be easily achieved by a reversal of stimulus (e.g., negative values). We select positive feedback due to the work in [43] which shows that recurrent excitation is the initial response of the reflex during perturbations; but, inhibition can also occur. Also note that the intrasegmental projections (dashed lines) would correct for interaction torques but are not needed in this robot study (see above and [9]).

**Figure 3:**
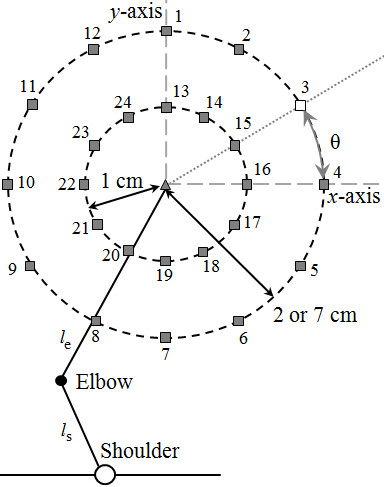
**Experimental setup for the first experiment.** Shows one set of concentric targets numbered 1 to 24 (squares) with an initial starting point (triangle). The *x* and *y* axes are illustrated in relation to the hand, and angle θ is given for an arbitrary target (#3 - white square). The target error is the magnitude distance between the target and the hand. These variables are used to solve eq. (1)-(5). Also observe the asymmetric geometric relationship between the upper arm, *l*_s_ = 12.5 cm, and the forearm, *l*_e_ = 22.5 cm.

The ASC is driven by an error vector (eq. 1) computed by the superior colliculus [26–28]; where *T* is the target coordinate, *H* is the hand coordinate, *x* and *y* represent the axes, and θ is the angle between the error vector in the horizontal plane and the *x*-axis (Figure 3). Previous studies demonstrated that the direction of motion (e.g., toward or away from the body) correlates with the activation of specific muscle groups, and that the projections of the error along the *x* and *y* directions (eqs. 2 and 3) correlate with activations in the shoulder and elbow actuators, respectively [3, 4, 9]. The vector is updated by vision using visual feedback (dashed-dot line, Figure 1) throughout the motion – also see [9]. The distributed error vectors (eq. 2 and 3) are then scaled by gains K_s_ and K_e_ (eq. 4 and 5) to tune motions based on limb geometric context and/or desired movement metrics (e.g., speed, duration, etc.) while preserving the appropriate multi-segment coordination. Then, the combined motor ‘go’ signal from each Mn drives the robot motors (Plants). Here, we demonstrate how these projections contribute to biomimetic control of a robotic arm, however future studies will demonstrate how these projections can be applied to artificial limbs by emulating the discharges of the superior colliculus.

12345

In Figure 1, the primary motor drive for each joint, *e*_*cs*_ and *e*_*ce*_, of the ASC is a “visually” perceived hand-to-target error vector [26–28, 44–49] in a plane weighted by tunable gains. The shared magnitude of the error vector effectively interconnects the two sides of the controller despite the absence of direct intrasegmental connections, while the trigonometric weightings are smooth and prevent the “discontinuous boundaries” discussed in [3]. Terms *e*_*cs*_ and *e*_*ce*_ are scaled by tuning gains K_s_ and K_e_.

It was shown previously [3, 4] that the magnitude of this error can suffice as a motor command for *both* the shoulder servo-motor, eq. (4), and elbow servo-motor, eq. (5), if given appropriate directional signs with respect to the rotation directions of each joint. That is, if the *y*-error is positive, then the arm will extend away from the body, whereas if it is negative it will bend inwards. Similarly, if the *x*-error is positive the arm will rotate clockwise, and vice-versa. This also agrees with evidence that the superior colliculus encodes stimuli via gain fields based on the magnitude and direction of perceived targets [26] – we assume *K*_*s*_ and *K*_*e*_ arise from this.

The projected errors (*e*_*cs*_, *e*_*ce*_) are then combined with the efference copy estimates (*e*_*fs*_, *e*_*fe*_) to define each plant’s motor drive as per Figures 1 and 2. The efference copy estimates are determined by the output of the models for each segment which predict the expected output of the actual plant. Recall, these models are represented by unique transfer functions which determine what is expected to result from a given motor input – eqs. (6) and (7). Moreover, it should be understood that visual feedback is not necessarily needed after the initial target selection. For example, if visual information is removed from the controller during a movement, the remaining efference model feedback loops would continue to react to a ‘step’ error. We refer to feedback at the spinal level as a ‘model’ in order to denote the presence of a reasonably accurate internal measure of the motor state. Realistically, it could just as easily rely on signals from proprioceptive or spindle reflexes in the biological case, or on segment sensors in the robot case.

In the controller (Figure 1), the sensory input gains, K_s_ and K_e_, modify the weight of error projections to the joints, and/or change the relative distribution of the error projections between joints. K_s_ and K_e_ are interpreted to regulate the input to the system much like projected scalings from the Colliculi that have been observed in the literature [26–28, 44–49] – i.e., discharges from reach-related neurons in the underlying layers of the superior colliculus. It is expected that these relative gains change both the kinematics and dynamics of the system, as expected from modulations in loops. All simulations of the controller were developed in Matlab (Mathworks, MA). Also see Appendix A for more information.

## Experimental paradigms

### Simple reaching (4 cm and 7 cm vectors)

*Target/arm locations* - The first experiment consists of a center-out reaching task to 12 targets (Figure 3). The hand will begin at the center position, reach to a target, move back to the starting position, then reach to another target, etc. The 12 targets are placed in a concentric circular pattern with uniform radial distances from the initial starting point – the large amplitude motion has a 7 cm radial target distance, while the smaller amplitude motion has a 4 cm radial distance. This setup will be used to evaluate the controller’s behavior in omni-directional motions for both small and larger reaches. The larger motions will be performed at a single set-point to highlight behavioral changes due to larger reaching motions. The small amplitude reaches are repeated using various robot arm geometries throughout the workspace to identify behavioral changes due to a changing set-point. In addition, the small amplitude motions will include a second concentric target circle with a 2 cm radius for comparison (Figure 3). Five sets of the smaller target patterns are distributed throughout the workspace. Note that this is not possible for large amplitude motions due to the size of the useable workspace. Therefore, each target set will have a different initial arm configuration while the *relative* target positions are all identical. The coordinates, in centimeters, of the initial hand positions for the reaching tasks are: (3.5, 29.75)_1_, (11.5, 275)_2_, (15, 20.75)_3_, (24.5, 12.75)_4_, and (25, 3.75)_5_. The shoulder joint is defined as the origin with co-ordinates (0, 0).

*Tuning path curvature* – For both small and large reaches, the motion is repeated whilst changing the gain ratio, K_s_/K_e_, until a user defined criteria for “hand path straightness” is attained for each target or directional motion. When tuning K_s_ and K_e_ relative to one another an incremental iteration will be implemented to slowly increase one gain relative to the other, and repeated through trial and error until the desired relationship is achieved. The objective is to determine whether tuning of the input error weight-ratio (K_s_/K_e_) is alone sufficient to achieve relatively straight trajectories. Therefore, theoretically optimal search algorithms (i.e., learning) are not required for this feasibility study. The unusual arm geometry (forearm length = 2× upper arm length) exacerbates the difficulty of producing straight movement trajectories for the end-point and is intentionally selected as a more challenging test-bed for this proof of principle. The user defined criteria for straightness is achieved if the amplitude of trajectory deviation orthogonal to the path is less than 0.15 cm (i.e., ~2.1% of reach amplitude) or if the iterative gain increments do not further straighten the curvature. Finally, the motion is considered to be complete when the hand is within 0.1 cm of the target, marking the end of the simulation. This, and other experiments, will also be compared to the motions achieved with trajectory planning theories, as described in [10] to serve as a reference for biological-like motions.

### Perturbations during large reaches (7 cm)

**Figure 4:**
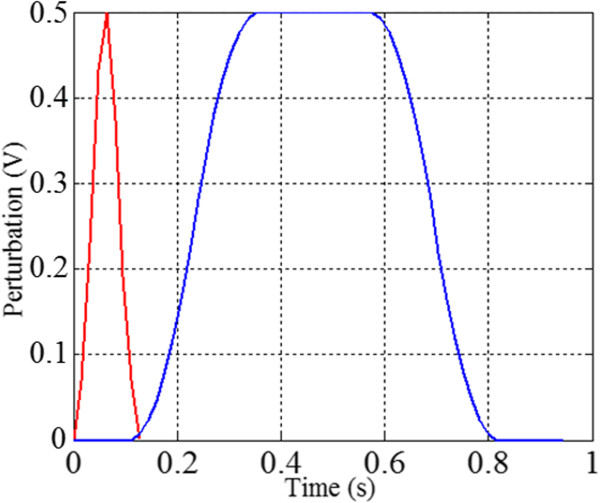
**Pulse and sustained perturbations.** The perturbation time plots are given for pulse perturbations (red line) and sustained perturbations (blue line). Notice that each perturbation reaches 0.5 Volts – this is equal to half the maximum possible motor drive for the plant in addition to the actual motor drive; e.g., if the plant is being driven internally by 0.5 volts, and a 0.5 volt perturbation is also applied, the total motor drive with perturbation will be 1 volts.

The second experiment examines larger reaching tasks with and without sudden position changes due to external perturbations. For this paradigm, perturbations are applied as additional voltage to the plants to change the motor outcome. Recall, that since the SID models of the plants include the dynamics of the multi-segment system, these perturbations do not represent a change in motor ‘go’ signals, but instead an external contribution which alters the dynamics. The results are comparable to our earlier findings for a multi-muscle system with perturbations induced in the biomechanics [9] and are thus assumed valid. This experiment is chosen in order to reproduce earlier perturbation experiments performed in [31]. We select an initial set-point at an arbitrary position and select a target location approximately 7 cm from the starting point. Here, the controller gain-ratios will be kept constant using values determined in experiment 1 for the relevant direction (Table 2, Cases 3, 7 and 11). The perturbations are applied as sudden and large voltages added to the control drives of both shoulder and elbow simultaneously and in the same direction (Figure 1). The efference models are assumed to rely on both expectant motor output and proprioception, thus the perturbations are applied to the plant at a time, *t*, and the model (efference copy loop) would see this change at time, *t + 1* (Figure 1). The efference models receive copies of all intended driving signals and any perturbation via proprioceptive feedback. Similar to [31], these perturbations are applied in two ways: (a) filtered impulses; and (b) sustained perturbations lasting ~0.5 s (see Figure 4). The magnitudes of these perturbations are equal to the half of the maximum expected servomotor input (0.5 V) in the absence of perturbations; hence these are very large perturbations. The motions with perturbations will be performed in 3 directions to illustrate consistency.

**Table 2:** Gain ratios to straighten motion paths to targets at location 3 experiment 1

***Target***	***K***_***s***_	***K***_***e***_	***Normalized***	***Target***	***K***_***s***_	***K***_***e***_	***Normalized***
			***K***_***s***_ ***: K***_***e***_				***K***_***s***_ ***: K***_***e***_
1	41	1	1 : 0.024	13	17	1	1 : 0.059
2	5	1	1 : 0.154	14	5	1	1 : 0.200
3	3	1	1 : 0.333	15	2	1	1 : 0.500
4	1	1.5	1 : 1.500	16	1	1	1 : 1.000
5	2.0625	1	1 : 0.485	17	2.0625	1	1 : 0.485
6	1	1	1 : 1.000	18	1	1	1 : 1.000
7	39	1	1 : 0.025	19	15.5	1	1 : 0.065
8	6	1	1 : 0.167	20	4.5	1	1 : 0.222
9	2.5	1	1 : 0.400	21	2	1	1 : 0.500
10	1	1.5	1 : 1.500	22	1	1	1 : 1.000
11	2.0625	1	1 : 0.485	23	2.0625	1	1 : 0.485
12	1	1	1 : 1.000	24	1	1	1 : 1.000

### Pursuit of moving target

The third experiment will determine how the controller behaves when reaching to a moving target which ‘jumps’ unexpectedly from one location to another. The initial *target position* is arbitrarily selected as (9.6, 17) cm and moves every 0.25 s in (+1, -0.5) cm coordinate jumps, until it reaches (12.6, 15.5) cm. In addition, the elbow and shoulder controller gains are set to a fixed ratio found in the same manner as in experiment 1. It is predicted that the hand will reach the final, stationary target in all cases, but with noticeable trajectory shifts each time the target jumps. Ultimately, the controller will be demonstrated as an adaptive system with “visual” integration allowing tracking in the presence of target position changes. Similar results can be demonstrated with any starting position as long as the target and its jumps remain visible and in the feasible workspace.

### Gains, target distance and hand speed

Finally, we will test the ASC’s capability to scale its motions based on desired movement criteria. As in the previous two experiments, the elbow and shoulder controller gains are set to the fixed ratio found acceptable in experiment 1, for the associated reach direction. This experiment will examine two concepts: (1) how hand path speed profiles change as a fixed target is placed further away from the hand while maintaining the same movement direction and controller parameters; (2) how a trajectory maintains its ‘straightness’ while increasing the hand-path speed for the same reach amplitude. Both experiments will start with hand positions at (9.6, 24) cm. The first paradigm will move to four targets located at (9.6, 17)_1_, (9.6, 19)_2_, (9.6, 21)_3_ and (9.6, 23)_4_ cm and will compare how the speed and curvature change for each motion using the default parameter set. The second paradigm will consist of a single 7 cm reach to (9.6, 17) cm, executed with different weights on K_s_/K_e_ (the ratio is preserved) to determine if the spatial trajectory is adaptive despite different hand path speed. Gain scaling will include 0.5*(K_s_/K_e_), 1*(K_s_/K_e_), 2*(K_s_/K_e_) and 4*(K_s_/K_e_), with reference ratio fixed at 15/1.

## Results

### Robot joint models

The parametric equations which model the robotic system’s shoulder, *M*_*s*_, and elbow, *M*_*e*_, joint are in equations 6 and 7. These equations represent the SID models described in the methods section for the robot apparatus and used as per Figure 1. These models describe the input/output relationship of the system and include the dynamics of the multi-segment system. Recall, during data acquisition for identification, the motor drives were limited to 0 ± 2 Volts (10 Volt robot maximum) to maintain slower speeds according to robot specifications, account for possible perturbations, and maintain a linear operating bandwidth.
67

### Simple reaching

**Figure 5:**
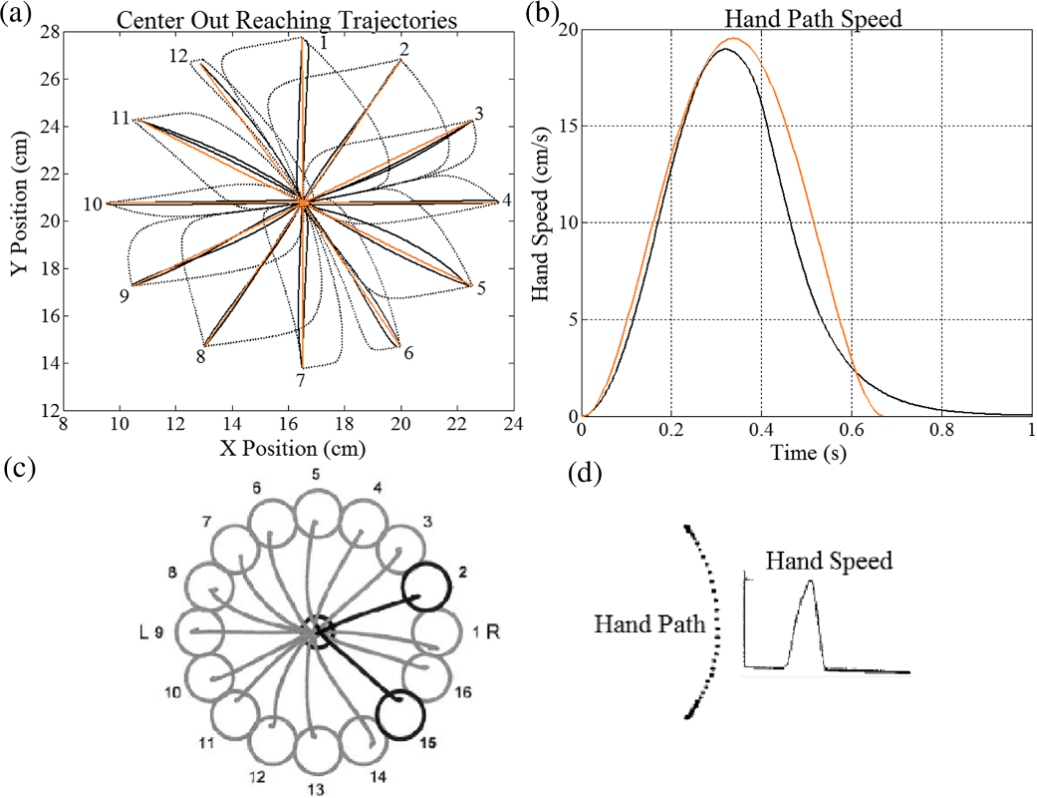
**Center out reaching motions. (a)** Center-out trajectories for 12 targets with untrained gains (dotted black line) and tuned gains (solid black line) as well as the trajectory planning path (orange line); The initial central hand position corresponds to case 3 in Figure 6. Also, the hand path speed is given in **(b)** for the motion to target 3; **(c)** Real center out reaching motions (right hand) as reported in [54]; **d)** point-to-point human reaching motion and the corresponding hand path speed as reported in [51].

**Figure 6:**
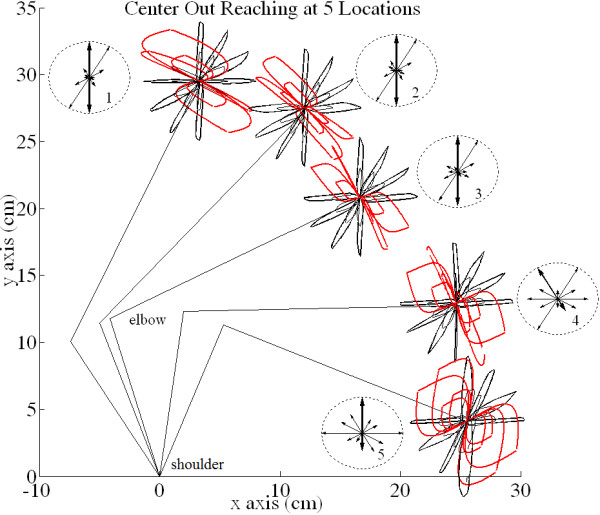
**Hand paths while reaching to 24 targets in 5 locations (4 cm & 2 cm distant).** The circular insets show the normalized magnitudes of gain ratios after tuning for each direction. They are plotted within a unit circle as arrows in the direction of the target from the hand. This demonstrates that gain tuning is dependent not only on target location relative to the hand, but also on the concurrent arm configuration, particularly here as the forearm approaches and crosses the horizontal line y = 12.5 cm (tangent to the elbow y-limit). Note that some trajectories are left untuned intentionally (red traces) – Figure 5 has already demonstrated that all directional motions can be tuned. Notice that these red trajectories naturally straighten or naturally become more curved as the configuration of the arm changes between target sets 1 through 5.

Figure 5 demonstrates how center-out movements can be straightened based only on iterative gain changes. Here, we compare highly curved motions (dotted black lines) with default gains (K_s_ = K_e_ = 1) and straightened motions (solid black lines) with tuned gains – we also provide a reference movement (orange lines) based on the trajectory planning method [10]. The curvature amplitude is measured using the longest orthogonal line from the reference to the curved motion.The untuned trajectories are heavily curved due to the large differences in the length of the forearm segment compared to the upper arm segment (~2:1 ratio). Also, each motion profile demonstrates its own curvature based on the direction of the target relative to the hand. When comparing default and tuned results, it is evident that significant straightening was achieved by simple gain tuning methods. Figure 5 shows that the hand path curvature amplitude to target 1 reduced by 2.65 cm (92.9%); the hand path curvature amplitude to target 2 reduced by 3.69 cm (97.8%); the hand path curvature amplitude to target 3 reduced by 1.53 cm (95%), etc. The tuned motions also have very similar motion profiles and hand path speeds (Figure 5b) when compared to the trajectory planning reference. Also, plots from biological reaching studies are shown in Figure 5c and d as a comparison. Figure 6 shows similar trends for tuned profiles in all five areas of the feasible workspace. Some of the trajectories are intentionally left untuned (red) to demonstrate how target positions and arm geometry naturally affect the shape of trajectory paths. Thus, as the set-point changes the trajectory can automatically straighten or widen without changing the gains (upper left, cases 3&4 in Figure 6). Similarly, the tuned gains for identical motion directions for two different arm configurations are different since arm geometry alters the motion characteristics.

Figure 6 provides insets next to each reaching task with associated gains shown as normalized vectors with respect to the largest ratio for the motion. The direction of each arrow coincides with the target directions relative to the hand for each motion, and a longer arrow indicates a larger gain ratio required for straightening. Table 2 quantifies this relationship by showing the normalized K_s_/K_e_ gain ratios for the straightened motions around central target position #3. These “optimum” gain ratios change in relation to the target patterns in the work space and with the arm’s initial geometry, as seen in the Figure 6 insets. Also to straighten trajectories, note that the important aspect of these (Ks, Ke) pairs is not their individual values, but rather the ratio between them.

### Trajectories in presence of perturbations while reaching

**Figure 7:**
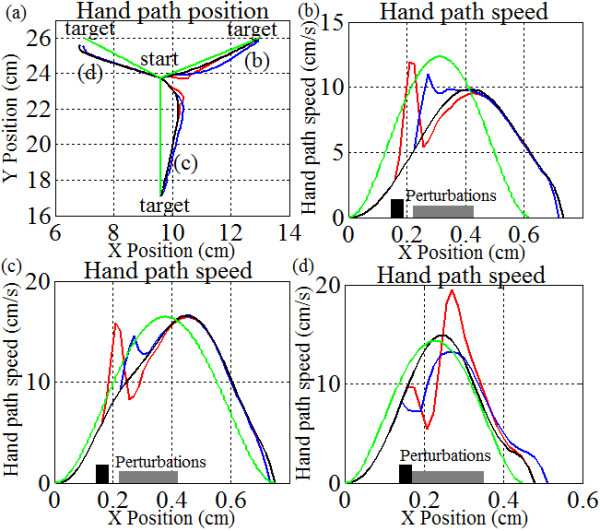
**Sensitivity of hand path and segment trajectories to applied perturbations.** Here, error vector magnitudes are tuned to produce a straighter path by emphasis of shoulder over elbow. The remaining parameters in the controller are identical to those in Figure 5. Notice that the trajectories for each hand path speed in **(b)**, **(c)** and **(d)** refer to the same path labels in **(a)**; solid black line (no perturbations), blue line (sustained perturbations), red line (pulse perturbations), green line (trajectory planning motion). Finally, observe that the speed profiles are not identical for each motion even though the initial hand-target distance is identical in magnitude. Also, motion **(d)** completes sooner – it is caused the geometric configuration and changes in motor drive of the shoulder/elbow with respect to one another. For more information see Figure 10. For each hand speed plot, the sustained perturbation time is shown as a dark grey bar, while the pulse perturbation time is shown as a black bar.

**Figure 8:**
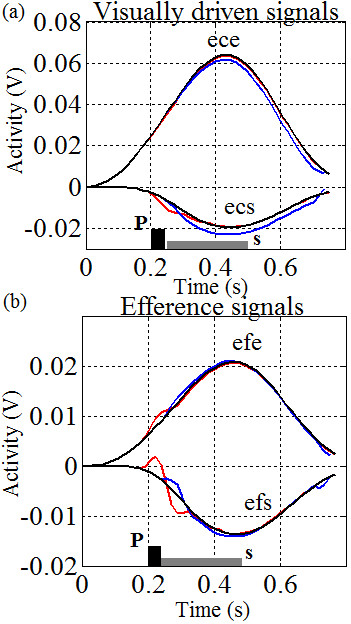
**Responses of the internal controller signals for motions in Figure** 7 **c.** Plots are shown without perturbations (black lines), with pulse perturbations (red lines) and sustained perturbations (blue lines). The control signals are given for the **(a)** visually driven error signals (*ece* and *ecs*); and **(b)** efference copy feedback (*efe* and *efs*) . Data in **(a)** are recorded after the spatial error ratio adjustments, as shown in Figure 1. Observe that there are noticeable variations between the profiles of the short-latency and long-latency responses to the perturbations as described in [30]. In particular, we can see a dominant role of the shoulder *ecs* for long-latency responses, and a more prominent role of the shoulder *efs* for short-latency response. The sustained perturbation (S) time is shown as a dark grey bar, while the pulse perturbation (P) time is shown as a black bar.

Figure 7 shows that the controller behaves differently when subjected to pulse and sustained perturbations. For example, when comparing the perturbed trajectories to the initial unperturbed movement (black line), there are noticeable differences in the corresponding hand path speeds. The motions with impulse perturbations (red lines) have shorter deviations from the unperturbed motion and larger hand path speed changes than the movement with sustained perturbations. For example, the hand path speeds during pulse perturbations change on average change by 9.5 cm/s with respect to the unperturbed motion, whilst the sustained perturbations cause speed changes of approximately 5 cm/s. Also, the perturbations cause a peak trajectory deviation of approximately 0.25 cm for both motions. Notice also that the perturbations can have accelerative effects (Figure 7b,c) or deceleration effects (Figure 7d) depending on the arm geometry and the direction of motion. These results show that the controller changes the hand path speeds change with the perturbation in order to maintain the trajectory profile. To add to this, it is of interest to note that the efference copy feedback is more dominant when responding to pulse perturbations (Figure 8b) whilst visual feedback is more dominant when responding to the sustained perturbations. This agrees with the delays expected from proprioceptive and visual feedback and is an important observation not described by earlier biomimetic controllers.Notice that motions in all three directions show similar perturbation effects for both trajectory and speed indicating that the phenomenon is not directionally dependent. Like before, the perturbations can either slow the hand-path speed (Figure 7d) or speed it up (Figure 7c). A reference with respect to the trajectory planning path is also given (green line) with similar speeds.

### Reaching to a moving target

**Figure 9:**
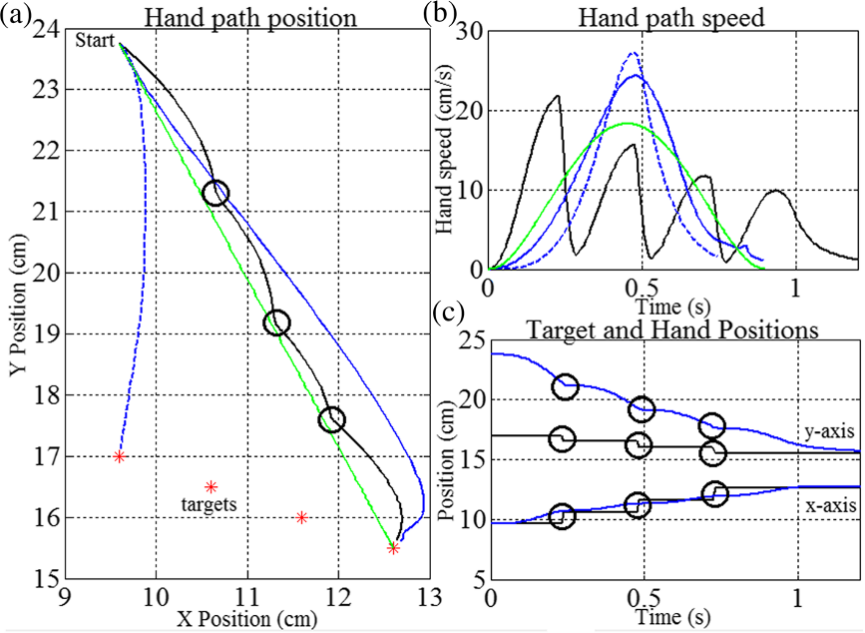
**Pursuing a jumping target. (a)** The target (red stars) begins at position (9.6, 17) cm and moves to (12.6, 15.5) cm. The hand trajectories (solid black line) noticeably change (at clear circles) each time the target jumps. The motion with a stationary initial target is shown (blue dotted line), as well as the motion directly to the final target position (solid blue line) and the trajectory planning motion (green line); **(b)** the corresponding hand path speeds are shown; **(c)** hand positions (blue lines) reaching to target positions in space (black lines) with respect to time given for *x* coordinate, and *y* coordinate. Notice that the multiple bell-curve speed profile caused by jumping targets matches those for movement via intermediate-targets, as reported in [10].

Figure 9 demonstrates that the controller is able to reach to moving targets without changes to the controller parameters or movement planning. Instead, the controller redistributes the motor commands based on each newly perceived target error (Figure 9a). In this case, the motions are executed with a fixed error gain ratio for a straight large reach in the direction of the last target (solid blue line) except for the motion to the original target position (blue dashed line). The hand path speed demonstrates bell-shaped peaks each time a new target is identified (Figure 9b). The timing of the trajectory changes are synchronized with the sudden *x*-*y* hand speed changes and with the target shifts (Figure 9b,c). The hand initially moves toward the target at (9.6, 17) cm but when the target is shifted to a new position, the trajectory also changes until the hand motion finally reaches the final target position at (12.6, 15.5) cm. The target positions with respect to time are given in Figure 9c; the solid line indicates the *x*-coordinate of the target and hand, while the dashed line indicates the *y*-coordinate. Figure 9b demonstrates that as the error vector changes with target shifts, multiple peaks occur as if reaching to multiple targets.

### Controller scaling of movement

**Figure 10:**
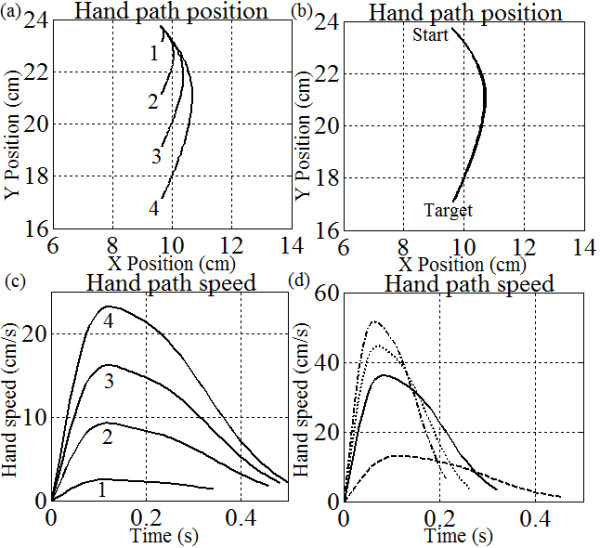
**Scaling of trajectory speed with reach amplitude and durations. (a)** increasing initial target distance in the same direction causes similar reach durations but larger hand path speed - the parameter set is fixed with K_s_/K_e_ = 15/1. The numbered trajectory paths in part **(a)** are matched with their hand path speed in part **(b)**. In **(c)** and **(d)**, we maintain a constant initial error vector while multiplying K_s_ and K_e_ by the same factor (preserve ratio); e.g., in **(d)** the solid black line’s K_s_/K_e_ ratio is 15/1 (case 4 in **(b)**), the dashed line is 7.5/0.5, the dotted line is 30/2, and the dash-dotted line is 60/4. All four conditions have identical spatial trajectories **(c)**, despite increases in path speed and concurrent decreases in reach duration **(d)**.

Possibly the most intriguing results are shown in Figure 10, where the hand path trajectories are all smooth, with single-peaked speed profiles. However, when the target distance from the hand is increased by uniform amounts (2 cm), the hand path speed peaks also increase in amplitude uniformly (by approx. 8 cm/s). Also, the time of movement remains similar. Therefore, Figure 10a-b demonstrates that movements of different amplitudes can be completed in the same time, because an increased error drive (i.e. loop gain) increases the speed of the motion as in any PD control system. The controller did this automatically in Figure 10a-b without any changes in parameters. Figure 10c-d, on the other hand, demonstrates that the controller can execute the same motion at different speeds. Notice that the hand path remains nearly identical in 9c, despite four different movement speeds and durations shown in 9d. This behavior is maintained up until the hand reaches a speed of approximately 60 cm/s. After this considerable oscillations can manifest (not shown) at the end-point. Regardless, tuning the temporal and spatial (curvature) properties of a movement can be done independently in this ASC. To the best of our knowledge this is the first biomimetic controller to demonstrate these abilities.

## Discussion

We demonstrate that the ASC is a versatile controller for goal-oriented reaching tasks and that its structure promotes adaptive control, responsive behavior, motion tunability, temporal and spatial scaling, and target tracking. To the best of our knowledge, this is the first biomimetic feedback-based controller that reaches all of these milestones. This preliminary study focused on the effects of gain tuning and how it affects motion behaviors. Novel findings of this research include:

- Gain distributions for the 2 segments control path curvature and their optimizations are dependent on target direction and limb geometry
- If a particular gain ratio is maintained, a reaching motion in any direction can be scaled to various peak speeds without greatly changing the spatial trajectory of the hand.

Such decoupling of hand kinematics from dynamics makes it a very adaptable controller for use in practical applications or in the study of reaching in primates. Future work will explore these gain fields for dynamic tuning and how they are learned over time for different directions and set-points.

### Trajectory profiles adjusted by error projection weights

In the center-out reaching experiments (Figures 5 and 6), it was demonstrated that the hand trajectories can be significantly straightened regardless of the target’s position relative to the hand, or the arm’s configuration (Figure 6). This was accomplished by tuning the *ratio* of error projection weights to each motor unit (or joint). The ratio for trajectory tuning is dependent on the direction of motion to the target as well as the geometrical configuration of the arm. Additionally, the distance to the target does not affect these ratios, so the same ratio can be used to tune all reaches in the same direction at various distances. For example, in Table 1 the same ratios are used to tune the 4 cm reaches and the 7 cm reaches for each direction in Figure 6. Note that this is an important observation, due to the asymmetric geometric properties of the robot arm; the robot forearm is twice as long as the upper arm, while the two segments are nearly equal for humans. This coincides with observations in [50] which suggest that biomechanical variations, even at the wrist, can affect the motion trajectories of the entire arm. Furthermore, the dynamics of the motion also change based on this gain manipulation. This represents an alternative interpretation for motor learning and trajectory formation than explicitly planned motion kinematics as discussed in [10]. For example, adjusting the gains for one segment relative to the other allows the controller to be scaled so that the parts behave differently relative to one another. In this study, the optimized gain-ratio was determined to be direction and set-point dependent which is reminiscent of the discharges from reach related neurons in the superior colliculus [26–28].

Notice that after tuning the majority of movement trajectories resemble those recorded in biological studies [10, 50–53]. They are defined by smooth, slightly curved motions with bell-shaped speed profiles. This is demonstrated in Figures 5, 7 and 9 when comparing the motion’s characteristics to the trajectory planning reference. Graham et al. [54] demonstrated that significant curvatures are present in untrained arm motions but are straightened with increased practice and/or joint stiffness. Recall, gain tuning in these experiments demonstrates the same progression from wide motions to straight motions using an incremental trial and error technique. Ultimately, the gain ratios proposed here could be interpreted in biology as changes in cell recruitment and/or in synaptic gains between error maps and spinal pre-motor circuits, as discussed in [6, 45].

### Compensation for perturbations

Since our controller operates on the basis of sensory-motor interaction, the system automatically adapts to the perturbations caused by sudden position changes. Figure 7 highlights this ability, where an initial position change due to a sudden mechanical perturbation causes the controller to compensate with force changes and sudden speed bursts. When the perturbation ends, the controller settles back onto its original path. Even though the trajectories are never explicitly planned, the motions in all three cases adhere to very similar profiles and the disturbed hand path decays back to the undisturbed trajectory. Recall, this ASC includes segment interaction, scalability, and sensory-specific network interactions. The ability of the ASC to quickly correct motions is a direct result of these relationships [18] since circuit parameters (Figure 2) could be modified based on proprioceptive changes or visual information. An observer on the outside might interpret these results as requiring pre-computation of desired trajectories but the same result is achieved here from the topology and built-in reflexes, not pre-planning. Also, recall that this behavior evolves independently from the pre-motor gains, *K*_*s*_ and *K*_*e*_.

### Reaching to moving targets

It is evident that the controller is able to reach moving targets (Figure 9) provided that the target does not ‘out-run’ the hand motion; i.e. remains in the controller bandwidth. Since the motion is based on perceived sensory stimuli [3, 4] the motor commands will change with visual target perception. Thus, motor pre-planning is not required. Due to the fact that sensory signals are responsible for driving the system, it is evident that the controller should be able to respond adequately to a variety of external effects. Without the need to plan or correct with a pre-computed motion, the spinal-like controller demonstrates an ability to adjust its motion plan based strictly on these sensory systems and to return to a default trajectory despite the absence of planning. This behavior is omitted from earlier biomimetic controllers, and it is unclear how planned or pre-programmed strategies could be modified fast enough to account for sudden target shifts or external limb perturbations. At this time, extensions to allow for visual and cognitive delays have not been included. This will be included in a future biological implementation of the controller.

The speed profiles in Figure 9b are also noteworthy. As the target shifts position, the ASC readjusts its motor commands and the speed profile demonstrates multiple bell-shaped curves. This is reminiscent of the multiple bell-shaped curves that are produced when movements appear to pass through intermediate (virtual) targets to avoid an obstacle or to build a more complex movement [10]. Our results show this could also be done without explicit planning, using only intermediate targets – visual or virtual. This is a unique behavior inherent to the ASC that is seldom seen in earlier biomimetic controllers. Again, recall that this behavior evolves independently from the gain levels, *K*_*s*_ and *K*_*e*_.

### Scaling of trajectory size and speed

Figure 10 demonstrates how gain changes in the ASC can scale the trajectory and speed of a motion. There is a consistent and smooth progression as the target distance is increased from the initial starting point. The controller supports reaches of similar duration to targets at different distances by increasing the hand path speed (Figure 10b) *without* any parametric changes, and without any loss of precision. This precision is maintained until the hand speed exceeds 60 cm/s (not shown), then it would experience oscillations. Figure 10c-d show that spatially identical hand motions can also be executed at increasing speeds by multiplying the error gain ratio by an appropriate factor (i.e., task urgency). This result also coincides with Gribble et al.’s work [55] which links arm stiffness with arm speeds and accuracy. In other words, multiplying the gain ratio in the ASC increases the combined motor output which increases the stiffness of the system and the speed of the motion. Notably, the ASC’s hand motion accuracy is unaffected by such speed increases, as opposed to earlier approaches such as the equilibrium point hypothesis which fails to perform well at high speeds [56, 57]. In any case, these results demonstrate that it is simple to scale the movement tasks without any need for pre-planning. Both dynamic and kinematic scaling are imbedded features of the spinal-like system, making it an adaptive controller for reaching various paradigms.

By linking these results to biology, an alternate hypothesis can be offered for movement control given the *structure* of the spinal circuits and their *separable* spatial and temporal sensitivity to selected parameters. A potential role for cortical or cerebellar projections onto the spinal system would be to modulate the gains of sensory afferents, rather than directly modulate planned trajectories. Examples include: i) projection strength of visual errors onto motoneural circuits that are modulated in the Frontal Eye Fields and superior colliculus by location on the map and by cognitive effects such as visual saliency or task instructions; or ii) the strength of projections from limb-based sensors (like spindles) onto interneurons and motoneurons that can be modulated by descending projections to the spinal cord and to spindle γ fibers. In addition, this controller demonstrates an imbedded reflex modulation similar to that seen in spinal motor centers [9, 58, 59]. In particular, sign changes modify the dominance of “extensors” and “flexors” whilst the magnitude of the gain affects the strength of the reflex. A recent publication also demonstrated that EMG patterns of the controller when applied onto systems with non-linear muscle approximations show biphasic and triphasic burst patterns as seen in biology [9]. Since the above controller architecture automatically generates human-like EMG profiles, it is expected that this controller can be applied to developed enhanced myoelectric control of artificial limbs or FES. This will be explored in more depth in future studies.

## Conclusion

The ASC presents a novel interpretation of how spinal-like circuits can simplify the planning task, so that instead of computing a trajectory, specific ‘modes’ for the spinal-like circuits can emerge via parameter and muscle selection. We have shown that this can happen automatically using the learned gain fields from the colliculi. The ASC also exhibits novel characteristics that are not included in earlier biomimetic controllers including an adaptive performance for multi-joint arm movements, and scalability for temporal and spatial motion tasks. Based on the results presented above, the ASC resembled biological reaching data found in the literature for various tasks despite using a non-biological robot arm, and can automatically change behavior based on sensory information. Also, adaptive behavior (e.g., tracking moving targets, perturbations, etc.) is available independently of the system gains and naturally evolves due to the structure of the controller. However, gains can also independently affect the characteristics of these motions. This is relevant in modern applications because it provides a tunable system that is reflexively responsive to stimuli. Future work on this study will examine the ASC’s application on a prosthetic limb and myoelectric stimulators.

## Appendix A

**Figure 11:**
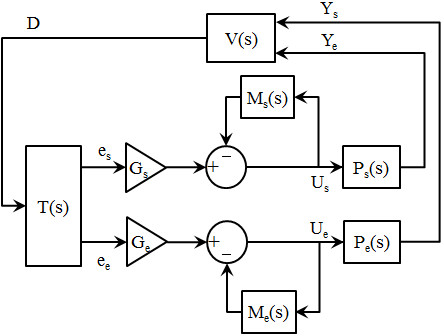
**Block diagram of the controller from Figure** 1 **.**

Consider the controller in Figure 1. If we assume that the actuators have linear characteristics and that we can lump agonist/antagonist actuators into a single bidirectional plant, the system can be simplified in block diagram form as shown in Figure 11.

Here, P(s) is the plant, M(s) is an approximation of the plant, G is a gain, T(s) is the error vector between the target and hand, V(s) represents the function which determines the hand spatial position, Y is the joint angle, U is the activation level, e is a projection of the hand-target error, and D is the hand position.

In order to determine the input/output characteristics of the system, we find a simple transfer function of the block diagram shown above. Here, we can present the transfer function as a relationship between the output (joint angle) and the input (input hand-target error), as follows:
A.1A.2A.3

Reorganizing equation (A.3) yields,
A.4

Therefore to simplify we acquire,
A.5

Substituting equation (A.5) into (A.2),
A.6

Similarly, since the two segments of the controller are identical,
A.7

**Figure 12:**
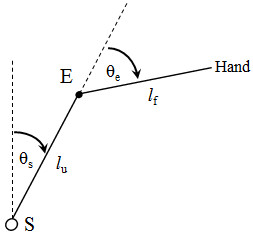
**Two link arm in space.** S is the shoulder joint, E is the elbow joint, *θ*_s_ is the shoulder angle, *θ*_e_ is the elbow angle, *l*_u_ is the length of the upper arm and *l*_f_ is the length of the forearm.

By changing the gain (G_e_ or G_s_) we affect the time constant of the system at the global level through the gain of the external error loops. This can have important implications for the controller since it can affect the segment contributions defined in equations (A.6) and (A.7), and with them the hand trajectory. Thus, since both segments have their own gain, the gains can be modified to affect both equations in the same proportion (i.e., scale time constants to remain uniform), or the gains may be configured to change the ratio of contributions in equations (A.6) and (A.7) with respect to (A.1) (i.e., scale time constants to be different in eq. (A.6) and (A.7)), thereby shaping trajectories. If the gains are properly selected, the controller will exhibit tunable behaviours. This is confirmed in Figure 5.Notice that equation (A.1) defines the hand position as some combination of eq. (A.6) and eq. (A.7). In reality, the relationship is a simple geometric solution (Figure 12).

Geometrically, determining the position of the hand is a simple calculation as shown in equations (A.8) and (A.9).
A.8A.9

Where *w*_*x*_ is the position of the hand on the *x*-axis, and *w*_*y*_ is the position of the hand on the *y*-axis. Thus, if equations (A.6) and (A.7) determine the joint positions based on the activation of the actuator, a simple transformation to coordinate space determines the hand position (*w*_*x*_, *w*_*y*_). With respect to Figure 11, equations (A.8) and (A.9) are analogous to V(s).
